# Accreditation as a management tool: a national survey of hospital managers’ perceptions and use of a mandatory accreditation program in Denmark

**DOI:** 10.1186/s12913-020-05177-7

**Published:** 2020-04-15

**Authors:** Louise A. Ellis, Anne Nicolaisen, Søren Bie Bogh, Kate Churruca, Jeffrey Braithwaite, Christian von Plessen

**Affiliations:** 1 Centre for Healthcare Resilience and Implementation Science, Australian Institute of Health Innovation, Macquarie University, Sydney, NSW 2109 Australia; 2 OPEN, Odense Patient Data Explorative Network, University of Southern Denmark and Odense University Hospital, DK-5000 Odense, Denmark; 3 Department of Clinical Research, University of Southern Denmark, Odense, Denmark; 4 Unisanté, Rue de Bugnon 44, 1011 Lausanne, CH Switzerland

**Keywords:** Certification/accreditation of hospitals, Quality management, Survey

## Abstract

**Background:**

This study aimed to examine managers’ attitudes towards and use of a mandatory accreditation program in Denmark, the Danish Healthcare Quality Program (Den Danske Kvalitetsmodel [DDKM]) after it was terminated in 2015.

**Methods:**

We designed a nationwide cross-sectional online survey of all senior and middle managers in the 31 somatic and psychiatric public hospitals in Denmark. We elicited managers’ attitudes towards and use of DDKM as a management using 5-point Likert scales. Regression analysis examined differences in responses by age, years in current position, and management level.

**Results:**

The response rate was 49% with 533 of 1095 managers participating. Overall, managers’ perceptions of accreditation were favorable, highlighting key findings about some of the strengths of accreditation. DDKM was found most useful for standardizing processes, improving patient safety, and clarifying responsibility in the organization. Managers were most negative about DDKM’s ability to improve their hospitals’ financial performance, reshape the work environment, and support the function of clinical teams. Results were generally consistent across age and management level; however, managers with greater years of experience in their position had more favorable attitudes, and there was some variation in attitudes towards and use of DDKM between regions.

**Conclusion:**

Future attention should be paid to attitudes towards accreditation. Positive attitudes and the effective use of accreditation as a management tool can support the implementation of accreditation, the development of standards, overcoming disagreements and boundaries and improving future quality programs.

## Background

Accreditation is concerned with the external assessment of organizations against designed, pre-approved standards. It has been a growing practice internationally for the last few decades [1]. Yet, the evidence to support its clinical and organizational value is inconsistent [2–6]. Although a systematic review of health sector accreditation suggested that it was able to promote change and professional development, the review also found evidence for substantial financial costs, and demands on staff time and other resources [7]. This variability in the evidence for accreditation in the international literature may also be in part due to differences in its implementation, timeframes, baseline levels, and how accreditation status is assigned.

The attitudes and perceptions of staff working within healthcare organizations are a key influence on the implementation of accreditation programs [8–11]. Previous research found that some staff value accreditation for creating organizational foundations for future quality improvement initiatives, [12] and for enhancing quality and organizational performance, while others perceived it as not worthwhile for patient care, and as bureaucratic and time-consuming [7, 11]. Such attitudes also vary by the type of staff member (e.g., doctors, nurses, managers), accreditation programs, and the context in which it is implemented [11]. A study on the Danish Healthcare Quality Program (Den Danske Kvalitetsmodel [DDKM]) found significant variations between physicians, nurses and other staff in their attitudes toward accreditation generally, and DDKM specifically [13]. In that study, however, managers comprised only a small portion of the sample and, hence, their attitudes were unable to be examined separately. Previous research on managers outside of the Danish context has found mixed results, with some studies identifying their negative views of accreditation (e.g., little benefit compared with time and cost), [14] and others positive views (e.g., promoting quality, good practices and uniting staff, [15] legitimizing their right to intervene in patient care) [16].

### Accreditation in Denmark

DDKM was a mandatory accreditation program for all Danish public hospitals, running from 2009 to 2015 led by the Danish Institute for Quality and Accreditation (IKAS). Its main purposes were to: enhance the quality of patient pathways; to improve the development of clinical, organizational and patient-perceived quality; and to make quality in the healthcare system visible.

DDKM was also meant to create learning and quality improvement through the ongoing assessment and feedback of each hospital’s results [17]. The first version of DDKM, launched in 2009, consisted of 104 standards, while the second version (from 2012) was reduced to 82 standards; with external surveys of the hospitals and revisions of the standards intended to take place every 3 years. IKAS’ surveyors assessed hospitals against standards for organization, patient pathways and disease-specific issues. Hospitals could either pass, pass with comments or fail based on their performance against the standards.

Over time DDKM received decreasing support from some politicians and healthcare professionals. It was criticized for the considerable demands it placed on resources, allegedly leaving less available time for direct patient care [18, 19]. After an intense political debate, the mandatory accreditation program for public hospitals was terminated in 2015, and the revised version of DDKM that was planned to launch in 2016 was not implemented [19]. In terminating the program, Danish politicians announced that DDKM for public hospitals had provided better quality of care, and it was time for a new program that would lead to reduced documentation and process control, and make room for quality improvement projects that made more sense to managers and staff [20].

Management’s attitudes toward accreditation are particularly important because they have been identified as a predictor of successful implementation of accreditation and other quality improvement initiatives [1, 10, 21–24]. Yet, there have been few rigorous studies investigating staff attitudes toward accreditation internationally, [11, 13] and even fewer with a focus on hospital management. In this regard, Denmark provides an important context through which to examine hospital management’s attitudes toward accreditation, because DDKM was promoted as a management tool, [25] but we know very little about how it was perceived and valued by those who were actually supposed to lead it. Further, the shift in policy and organizational processes brought about by the unexpected decision to abandon DDKM has provided a unique opportunity for reflection and adaptation among hospital management in Denmark. As a result, the aims of this study were to investigate managers’ attitudes towards and use of DDKM as a management tool.

## Method

### Study design

The study employed a nationwide cross-sectional survey of managers’ attitudes toward DDKM, asking about their perceptions of the program, and the purposes for which they had used it. Data for the study was collected at all public somatic and psychiatric Danish hospitals from March to June 2016.

### Survey population and administration

All senior and middle managers working in Danish public hospitals were invited to participate through an invitation sent to their work email address (*n* = 1095). On average, six senior managers and 29 middle managers were invited to participate per hospital. The email included a link to a SurveyXact™ online version of the survey. Participation was voluntary and anonymous, with no financial incentives being offered to enhance enrolment. For this study, senior managers included staff in either executive or upper-level leadership positions (e.g., hospital, nursing and medical directors, as well as heads of centers). Middle managers included staff reporting to senior management and having lower level management report to them (e.g., senior nurses, senior physicians, senior therapists and senior service managers).

### Survey design

The survey was designed following a review of national and international literature on accreditation. The purposes and standards of DDKM were considered, and items were constructed to elicit managers’ attitudes towards the accreditation program and how they had used the quality model in their leadership. Three of the authors (AN, SB, CvP), with international expertise on accreditation and quality improvement in healthcare, designed the survey with regard to the identification of four key themes relevant to the Danish context (i.e., Organisation of Care; Contribution to Outcomes; Professional Areas; and Management and Cooperation), and then they and JB reviewed and provided feedback on the wording of items mapping onto each of these four themes. Each item was translated forward to English and backward to Danish according to guidelines proposed by Beaton and colleagues [26]. The survey was pilot tested with a small group of managers and researchers within the quality improvement field (*n* = 10), to check for possible misinterpretations of questions, instructions and response format. Only minor adjustments were made to the final version of the survey, including a more detailed written introduction to the survey and minor amendments to the wording of some of the items to improve clarity.

The final survey consisted of 19 items designed to elicit managers’ attitudes towards DDKM as a management tool and 19 items on how managers used DDKM. These items were rated on a 5-point Likert scale ranging from 1 (Strongly Disagree) to 5 (Strongly Agree). Internal consistency estimates demonstrated good reliability for each of the key theme areas, ranging from .80 (Attitudes: Professional Areas items) to .94 (Use: Management and Cooperation items). All items are presented in Supplementary file 1. Respondents were also asked about their age, hospital site, job title, years in current position, and participation in first and/or second version of DDKM.

### Data analysis

Descriptive statistics were performed as mean values and standard deviations (SD). Linear regression was used to examine differences in age, years in current position, and management level and region on each of the survey items. Five sets of dummy variables were initially created and examined for the categorical measure of region. For brevity, the results for all five of these dummy variables are not presented here, but are available on request. In the regression analyses, ordinal categorical variables with five or more levels were treated as continuous variables [27]. All data were analyzed via IBM’s SPSS Statistics Version 24 and conducted at the 0.05 significance level.

## Results

Of 1095 eligible managers, 536 (49%) across all 31 hospitals participated in the survey. A substantial proportion of respondents had been in their current position for more than 3 years (69%), and the majority had participated in either the first (82%) or second (95%) version of DDKM (see Table 1). More than four-fifths of the respondents were middle managers (82%).

**Table 1:** Demographic characteristics of the study sample

	***n***	***%***
Age		
< 45 years	38	8.8
46–50 years	41	9.5
51–55 years	108	24.9
56–60 years	147	33.9
61–65 years	83	19.2
> 66 years	16	3.7
Years in current position		
< 1 year	32	6.0
1–3 years	133	24.8
4–6 years	109	20.3
7–9 years	91	17.0
> 9 years	171	31.9
Management level		
Senior management	100	18.7
Middle management	436	81.3
Participation in accreditation		
First version of DDKM	441	82.3
Second version of DDKM	509	95.0
Other forms of accreditation	357	66.6

Note. The sum for Age does not add up to the total (*N* = 536) because some values were missing

### Attitudes towards DDKM as a management tool

Respondents’ evaluations of DDKM were most favourable in terms of the support it provided in standardizing care processes (mean = 3.82), improving patient safety (mean = 3.81), and clarifying responsibility in the organization (mean = 3.68), with more than two thirds of respondents agreeing or strongly agreeing that DDKM supports them in these three areas (75, 73, and 68%, respectively, see Table 2). These positive results held uniformly across age, years in role, and management level. Approximately half of the respondents agreed or strongly agreed that DDKM provides support for increasing quality of patient care by identifying problems (50%; mean = 3.40) and in improving care (42%; mean = 3.27).

**Table 2:** Hospital managers’ attitudes towards and use of DDKM

Item	Attitudes towards DDKM				Use of DDKM			
	n	Mean	SD	% Agree	n	Mean	SD	% Agree
Standardize care processes	536	3.82	0.90	74.6	484	3.75	0.93	71.0
Increase quality of care by identifying problems	536	3.40	0.96	50.4	488	3.39	1.00	50.2
Improve the organization of care	536	3.18	0.97	39.9	490	3.34	1.01	49.0
Improve care	535	3.27	0.85	42.4	485	3.16	0.98	39.6
Improve patient safety	535	3.81	0.84	72.6	499	3.69	0.93	70.5
Improve work environment	535	2.82	0.92	20.8	501	2.76	0.93	19.2
Improve quality in my organization/department	535	3.63	0.92	63.1	505	3.56	0.97	61.6
Increase patient satisfaction	535	2.96	0.88	23.9	488	2.94	0.91	24.4
Enhance the professional development of the staff	532	3.14	0.99	38.0	499	3.17	1.03	41.7
Enhance feedback from the staff to the management	532	2.95	0.92	26.3	495	3.00	0.97	29.5
Clarify roles in the organization	518	3.52	0.91	60.3	493	3.47	0.95	58.4
Clarify responsibility in the organization	518	3.68	0.88	68.1	493	3.60	0.92	65.9
Improve the organizations financial performance	518	2.64	0.92	15.4	488	2.70	0.97	18.9
Improve the reputation of my organization	518	3.09	0.90	32.8	485	3.05	1.00	33.6
Support the organization of administrative teams	518	2.87	0.88	23.1	496	2.89	0.97	25.7
Support the function of clinical teams	518	2.87	0.84	22.0	484	2.84	0.91	23.0
Support the function of interdisciplinary teams	518	2.97	0.91	27.8	485	2.99	0.96	31.3
Support the internal cooperation by promoting the same norms and speech	518	3.50	0.89	58.5	490	3.50	0.97	60.2
Support the cooperation with healthcare staff across sectors	518	3.36	0.90	49.4	486	3.07	0.98	35.4

On the other hand, respondents were least favourable about the support DDKM provided in improving the organization’s financial performance (mean = 2.64), improving the work environment (mean = 2.82), supporting the function of clinical teams (mean = 2.87) and supporting the organization of administrative teams (mean = 2.87). Across these items, less than one-quarter of respondents agreed or strongly agreed that DDKM supports them in these ways (15, 21, 22 and 23%, respectively). Again, respondents’ views for these items held uniformly across age, years in role, and management level.

There were significant differences between management groups, and years of experience in current role, and regions for certain other attitude items (See Table 3). First, senior managers (mean = 3.43) were more positive than middle managers (mean = 3.23) in their attitudes towards DDKM’s support in improving care (*p* = .039). Further, years in role was a significant predictor for five of the attitude items, with managers with increased years of experience being more positive about the support DDKM provided in increasing quality of care by identifying problems (*p* = .022), improving the organization of care (*p* = .007), increasing patient satisfaction (*p* = .009), enhancing feedback from staff to management (*p* = .040), and improving the reputation of their organization (*p* = .046).

**Table 3:** Simple regression analysis for variables predicting attitudes towards and use of DDKM

	Attitudes towards DDKM			Use of DDKM		
	*β*	t	95% CI	*β*	t	95% CI
Standardize care processes						
Age	−0.03	−.60	−.02 – .01	−.16	−3.04**	−.04 – −.01
Years in role	.07	1.57	-.01 – .11	−.02	−.42	−.08 – .05
Management level	−.08	− 1.92	−.39 – .01	−.04	−.84	−.31 – .13
Region	.18	4.13**	.18 – .50	.18	3.89**	.17 – .53
Increase quality of care by identifying problems						
Age	.00	−.03	−.02 – .01	−.06	− 1.14	−.03 – .01
Years in role	.10	2.31*	.01 – .14	.06	1.18	−.03 – .12
Management level	−.05	− 1.13	−.34 – .09	−.02	−.33	−.27 – .20
Region	.14	3.30**	.12 – .46	.16	3.41**	.14 – .52
Improve the organization of care						
Age	−.02	−.41	−.02 – .01	−.10	− 1.94	−.03 – .00
Years in role	.11	2.61**	.02 – .15	.01	.11	−.07 – .08
Management level	.03	.71	−.14 – .29	.02	.33	−.20 – .28
Region	.09	2.07*	.01 – .36	.16	3.38**	.14 – .52
Improve care						
Age	.01	.17	−.01 – .02	−.06	− 1.19	−.03 – .01
Years in role	.06	1.30	−.02 – .16	.02	.41	−.06 – .09
Management level	−.09	−2.14*	−.41 – -.02	−.01	−.21	−.25 – .20
Region	.14	3.14**	.09 – .41	.14	2.89**	.09 – .46
Improve patient safety						
Age	−.01	−.24	−.01 – .01	−.05	− 1.01	−.02 – .01
Years in role	.06	1.28	−.02 – .09	.01	.14	−.06 – .07
Management level	−.08	− 1.70	−.35 – .03	−.05	− 1.01	−.33 – .11
Region	.20	4.68**	.21 – .50	.15	3.24**	.12 – .47
Improve work environment						
Age	.00	−.02	−.01 – .01	−.07	−1.39	−.03 – .01
Years in role	.03	.70	−.04 – .08	.03	.57	−.05 – .09
Management level	−.03	−.72	−.28 – .13	−.02	−.46	−.27 – .17
Region	.12	2.68**	.06 – .39	.10	2.19*	.02 – .38
Improve quality in my organization/department						
Age	−.04	−.77	−.02 — .01	−.07	− 1.33	−.03 – .01
Years in role	.00	.01	−.06 – .06	.04	.74	−.04 – .10
Management level	−.02	−.42	−.25 – .16	−.02	−.43	−.28 – .18
Region	.16	3.71**	.14 – .47	.18	3.76**	.17 – .54
Increase patient satisfaction						
Age	.00	.07	−.01 – .01	−.07	− 1.35	−.03 – .01
Years in role	.11	2.47*	.02 – .13	.02	.33	−.05 – .08
Management level	.03	.68	−.13 – .26	−.02	−.33	−.25 – .18
Region	.12	2.77**	.06 – .38	.14	3.03**	.09 – .44
Enhance the professional development of the staff						
Age	.03	.62	−.01 – 0.2	−.03	−.62	−.02 – .01
Years in role	.06	1.41	−.02 – .11	.00	.03	−.07 – .08
Management level	.00	.06	−.21 – .23	.03	.56	−.17 – .31
Region	.14	3.31**	.12 – .47	.11	2.43*	.05 – .44
Enhance feedback from the staff to the management						
Age	.06	1.28	−.01 – .02	−.04	−.76	−.02 – .01
Years in role	.09	2.05*	.00 – .13	−.00	−.02	−.07 – .07
Management level	−.08	− 1.85	−.40 – .01	−.08	− 1.73	−.42 – .03
Region	.15	3.48**	.13 – .45	.18	3.80**	.17 – .53
Clarify roles in the organization						
Age	−.03	−.64	−.02 – .01	−.01	−.16	−.02 – .02
Years in role	.03	.57	−.04 – .08	.01	.13	−.07 – .07
Management level	−.01	−.12	−.21 – .19	.01	.14	−.21 – .24
Region	.11	2.45*	.04 – .36	.16	3.38**	.13 – .50
Clarify responsibility in the organization						
Age	.03	.68	−.01 – .02	−.02	−.40	−.02 – .01
Years in role	.05	1.04	−.03 – .09	.02	.49	−.05 – .08
Management level	.03	.69	−.13 – .26	−.01	−.10	−.23 – .21
Region	.15	3.44**	.12 – .43	.19	3.99**	.18 – .53
Improve the organizations financial performance						
Age	−.01	−.24	−.02 – .01	−.07	−1.24	−.03 – .01
Years in role	.04	1.00	−.03 – .09	.04	.89	−.04 – .10
Management level	.00	.03	−.20 – .20	.01	.24	−.20 – .26
Region	.07	1.66	−.03 – .30	.08	1.63	−.03 – .34
Improve the reputation of my organization						
Age	.02	.36	−.01 – .02	−.04	−.74	−.02 – .01
Years in role	.09	2.00*	.00 – .12	.04	.75	−.05 – .10
Management level	.02	.42	−.16 – .24	−.04	−.74	−.32 – .15
Region	.06	1.27	−.06 – .26	.09	1.87	−.01 – .37
Support the organization of administrative teams						
Age	.07	1.48	−.00 – .02	.05	.92	−.01 – .02
Years in role	.04	.94	−.03 – .09	.08	1.59	−.01 – .13
Management level	.02	.51	−.14 – .24	.05	1.08	−.10 – .35
Region	.06	1.46	−.04 – .27	.10	2.11*	.01 – .39
Support the function of clinical teams						
Age	−.02	−.39	−.02 – .01	−.03	−.53	−.02 – .01
Years in role	.04	.87	−.03 – .08	.06	1.32	−.02 – .11
Management level	.01	.18	−.17 – .20	.07	1.44	−.06 – .37
Region	.04	.85	−.09 – .22	.05	.96	−.09 – .26
Support the function of interdisciplinary teams						
Age	.04	.86	−.01 – .02	−.01	−.17	−.02 – .02
Years in role	.06	1.45	−.02 – .10	.06	1.30	−.02 – .12
Management level	.05	1.06	−.09 – .31	.08	1.60	−.04 – .41
Region	.08	1.92	−.00 – .32	.10	2.04*	.01 – .38
Support the internal cooperation by promoting the same norms and speech						
Age	−.01	−.19	−.02 – .01	−.06	−1.04	−.03 – .01
Years in role	.08	1.80	−.01 – .11	.03	.67	−.05 – .09
Management level	−.00	−.09	−.20 – .19	−.03	−.55	−.29 – .16
Region	.11	2.58*	.05 – .36	.12	2.44*	.04 – .42
Support the cooperation with healthcare staff across sectors						
Age	.02	−.34	−.02 – .01	−.11	−2.05*	−.03 – -.00
Years in role	.05	1.04	−.03 – .09	−.01	−.30	−.08 – .06
Management level	−.06	−1.25	−.32 – .07	.02	.39	−.18 – .27
Region	.12	2.68**	.06 – .38	.06	1.34	−.06 – .31

* *p* < .05, ** *p* < .01; Management level (senior management = 0, middle management = 1); Region (The Capital Region of Denmark = 1, Region Zealand = 1, Region of Southern Denmark = 1, Central Denmark Region = 0, The North Denmark Region = 0)

From an analysis of regional differences, it was evident that the results for regions 4 and 5 (i.e., Central Denmark Region and The North Denmark Region) were significantly lower than the other three regions, so a new dummy variable was created to reflect this (i.e., The Capital Region of Denmark = 1, Region Zealand = 1, Region of Southern Denmark = 1, Central Denmark Region = 0, The North Denmark Region = 0) with results for this dummy variable presented in Tables 3 and 4. As shown in Table 3, managers from the Capital Region of Denmark, the Region Zealand and Region of Southern Denmark reported more positive attitudes towards DDKM for the majority (14/19 = 74%) of attitude items.

### Participants’ use of DDKM as a management tool

A similar pattern of results emerged for managers’ use of DDKM (see Table 2). Respondents reported that they were most likely to have used DDKM as a management tool to standardize care processes (mean = 3.75), improve patient safety (mean = 3.69) and clarify responsibility in the organization (mean = 3.60). Again, more than two-thirds of respondents agreed or strongly agreed, in this case, that they used DDKM for these purposes (71, 71 and 66%, respectively, see Table 2).

Respondents were least likely to have used DDKM as a tool to improve the organization’s financial performance (mean = 2.70), improve the work environment (mean = 2.76), support the function of clinical teams (mean = 2.84), and support the organization of administrative teams (mean = 2.89). Less than one-quarter of respondents agreed or strongly agreed that they used DDKM for these purposes (19, 19, 23 and 26%, respectively).

Age was a significant predictor for two of the items, with younger managers being more likely to have used DDKM to standardize care processes (*p* = .010) and to support the cooperation of healthcare staff across sectors (*p* = .011). Again, region was a significant predictor for most of the “use” items, with managers from the Capital Region of Denmark, the Region Zealand and Region of Southern Denmark being more likely to report that they had used DDKM for the purposes listed (see Table 3). There were no significant effects for management level or time in role for any of the “use” items.

## Discussion

This is the first large-scale study to focus on hospital manager’s attitudes towards and use of the Danish healthcare accreditation program, and one of the very few internationally. Overall, attitudes towards DDKM were more positive than negative, though there was considerable variation across items. In general, background characteristics such as age and management level explained very little of the differences in attitudes towards accreditation; a finding that is consistent with previous research [13]. However, experience was a significant predictor for five of the attitude items, with managers with greater years of experience in their current role reporting more favourable attitudes. Furthermore, there were significant differences in attitudes between regions, which may be due to regional leadership or diversity in experience with accreditation prior to the roll-out of DDKM. Two of the three regions that had more positive attitudes also had prior experience with international accreditation programs (e.g., Joint Commission), which may, at least in part, account for their more positive results.

Over 70% of respondents agreed that they had used DDKM for standardizing care processes and for improving patient safety. Indeed, the design of DDKM corresponds well with standardization and safety work. At the same time, only half of the respondents had used DDKM to increase the quality of care by identifying problems, which may suggest a preference for quality regulations that are not based on *controlling* quality but rather on *improving* quality. Further, almost two-thirds of respondents agreed that they had used DDKM for improving quality in their hospital or department, so while standards may be useful for quality improvement, DDKM might have simply had too many, and not locally rooted, standards.

On the other hand, the most negative ratings towards DDKM related to its ability to improve hospital’s financial performance, with less than one in five managers in the sample agreeing they had used it for this purpose. Indeed, the cost of accreditation programs has been raised as a consistent concern in previous healthcare accreditation research, [7, 11] with some questioning whether the high costs associated with accreditation is an appropriate use of time and resources. Mitigating this, Mumford et al. (2015) found that accreditation in Australia costs between 0.03 and 0.60% of total hospital operating budgets, averaged over a four-year cycle [28].

Respondents’ most negative attitudes concerned DDKM’s ability to re-shape the work environment; improve the organization’s support for the function of clinical teams; enhance the organization of administrative teams; and support the functioning of interdisciplinary teams. These are all areas with standardized processes in DDKM, and it is interesting that so many respondents gave less favorable responses with several items concerning *Management and Cooperation*. One explanation could be that DDKM focused on processes rather than outcomes. For example, although there are standards for how to deliver feedback from staff to management, this may not be practiced in everyday work so much as used only for accreditation purposes. Another reason may be the risk of a shift in managers’ focus from identifying areas for improvement, to focusing on how to fulfill standards [29]. However, more than half of the respondents used DDKM to clarify responsibilities in the organization; and to support internal cooperation by promoting sharing of knowledge and the same norms and speech. The latter is probably associated with the standardization of processes leading to the allocation of responsibility and identifying and describing required knowledge.

Overall, the study found that less than half of all managers had used DDKM to improve care, highlighting a concern regarding the increased processes and structures as detracting from a focus on patients. Indeed, two previous Danish reports have suggested that clinical staff find it difficult to acknowledge patients’ preferences due to the standardization of care brought about through accreditation [18] and view DDKM’s primary focus as on management and organization, rather than patients [19]. This creates a schism since several standards in DDKM intended to emphasize patient-centered care risk emphasizing greater levels of provider-oriented care. The managers’ task to implement DDKM may have therefore been impeded by the structure of the predefined standards, which might not fit with core values for staff with direct patient contact. In contrast to these negative attitudes with regards to DDKM’s capacity to realize improvements in care, recent Danish studies showed that fully accredited hospitals performed better on a range of indicators than partially-accredited hospitals [5, 6, 30]. It is possible, therefore, that such broadly-based associations may be difficult for management staff to recognize in their daily work with patient care as a core. In the present study, senior managers were significantly more positive than middle managers in their attitudes towards DDKM as a support tool in improving care, so it is possible that senior managers may be more able to make these broad-based associations.

As to limitations, this study is cross-sectional and an attitude survey, with the potential for self-selection bias. We cannot rule out that the non-respondents were different from the respondents, as information on non-respondents was not available. At the region level, all five regions are well represented and only two hospitals in the country were not represented at the senior level, and only one hospital at the middle level. In addition, the study enrolled half the senior and middle-level managers in the country; few national studies achieve this level of participation [31].

## Conclusions

In conclusion, both middle and senior managers reported strengths of accreditation. Accreditation was found most useful in standardizing processes, improving patient safety and clarifying responsibilities in the organization, yet it did not improve the work environment, support the function of teams or improve the financial performance of their hospital.

Accreditation was perceived somewhat useful for identifying problems, improving care and improving safety, yet it reportedly did not enhance the work environment or support clinical or administrative teams. Perhaps managers did not succeed in communicating the advantages of DDKM. More experienced managers and managers from regions with a history of international accreditation programs favored DDKM, possibly indicating that experience contributes to a better understanding of accreditation’s value and use. This might also reflect different generations of managers, and different attitude paradigms towards accreditation. Overall, positive attitudes and the effective use of accreditation as a management tool can support the implementation of accreditation, the development of standards, overcoming disagreements and boundaries, as well as improving future quality programs. Prior to rolling out a new accreditation program, it is important to engage hospital managers and foster positive attitudes through education and consultation.

## Supplementary information

**Additional file 1.** DDKM Attitude and Use Items.

## Data Availability

All data generated or analysed during this study are included in this published article: the technical appendix, statistical code and dataset are partially available in the provided appendices. The datasets used and/or analysed during the current study are available from the corresponding author on reasonable request.

## Abbreviations

DDKM: Danish Healthcare Quality Program (Den Danske Kvalitetsmodel)
IKAS: Danish Institute for Quality and Accreditation
