# Supplementary material for: Accreditation as a management tool: a national survey of hospital managers’ perceptions and use of a mandatory accreditation program in Denmark
Source: BMC Health Serv Res. 2020 Apr 15;20:306. doi: 10.1186/s12913-020-05177-7 (PMC7158040; doi:10.1186/s12913-020-05177-7)
Supplement: Supplementary file 1 — Additional file 1. DDKM Attitude and Use Items. [file 12913_2020_5177_MOESM1_ESM.docx]

**Supplementary File 1**

**DDKM Attitude and Use Items**

All items are rated on a five-point Likert Scale (1, strongly disagree; 5, strongly agree).

*Attitudes towards DDKM*

Managers´ attitudes towards DDKM as a management tool to:

*Organisation of Care*

1. standardize care processes
2. increase quality of care by identifying problems
3. improve the organization of care
4. improve care

*Contribution to Outcomes*

1. improve patient safety
2. improve work environment
3. improve quality in my organization/department
4. increase patient satisfaction

*Professional Areas*

1. enhance the professional development of the staff
2. enhance feedback from the staff to the management

*Management and Cooperation*

1. clarifying roles in the organization
2. clarifying responsibility in the organization
3. improve the organizations financial performance
4. improve the reputation of my organization
5. support the organization of administrative teams
6. support the function of clinical teams
7. support the function of interdisciplinary teams
8. support the internal cooperation by promoting the same norms and speech
9. support the cooperation with healthcare staff across sectors

*Use of DDKM*

Managers’ use of DDKM as a management tool to:

*Organisation of Care*

1. standardize care processes
2. increase quality of care by identifying problems
3. improve the organization of care
4. improve care

*Contribution to Outcomes*

1. improve patient safety
2. improve work environment
3. improve quality in my organization/department
4. improve patient satisfaction

*Professional Areas*

1. enhance the professional development of the staff
2. enhance feedback from the staff to the management

*Management and Cooperation*

1. clarifying roles in the organization
2. clarifying responsibility in the organization
3. improve the organizations financial performance
4. improve the reputation of my organization
5. support the organization of administrative teams
6. support the function of clinical teams
7. support the function of interdisciplinary teams
8. support the internal cooperation by promoting the same norms and speech
9. support the cooperation with healthcare staff across sectors
